# Achieving equitable uptake of handwashing and sanitation by addressing both supply and demand-based constraints: findings from a randomized controlled trial in rural Bangladesh

**DOI:** 10.1186/s12939-020-01353-7

**Published:** 2021-01-06

**Authors:** Sarker Masud Parvez, Musarrat Jabeen Rahman, Rashidul Azad, Mahbubur Rahman, Leanne Unicomb, Sania Ashraf, Momenul Haque Mondol, Farjana Jahan, Peter J. Winch, Stephen P. Luby

**Affiliations:** 1 Environmental Intervention Unit, Infectious Diseases Division, International Centre for Diarrhoeal Disease Research, Bangladesh (icddr,b), Dhaka, Bangladesh; 2 University of Pennsylvania, Philadelphia, PA USA; 3 Department of Statistics, University of Barishal, Barishal, Bangladesh; 4 Social and Behavioral Interventions Program, Department of International Health, Johns Hopkins Bloomberg School of Public Health, Baltimore, MD USA; 5 Division of Infectious Diseases and Geographic Medicine, Stanford University, Stanford, CA USA

**Keywords:** WASH benefits, Uptake, Equity, Wealth quintiles, Sanitation, Handwashing

## Abstract

**Background:**

Supply driven programs that are not closely connected to community demand and demand-driven programs that fail to ensure supply both risk worsening inequity. Understanding patterns of uptake of behaviors among the poorest under ideal experimental conditions, such as those of an efficacy trial, can help identify strategies that could be strengthened in routine programmatic conditions for more equitable uptake. WASH Benefits Bangladesh was a randomized controlled efficacy trial that provided free-of cost WASH hardware along with behavior change promotion. The current paper aimed to determine the impact of the removal of supply and demand constraints on the uptake of handwashing and sanitation behaviors across wealth and education levels.

**Methods:**

The current analysis selected 4 indicators from the WASH Benefits trial— presence of water and soap in household handwashing stations, observed mother’s hand cleanliness, observed visible feces on latrine slab or floor and reported last child defecation in potty or toilet. A baseline assessment was conducted immediately after enrolment and endline assessment was conducted approximately 2 years later. We compared change in uptake of these indicators including wealth quintiles (Q) between intervention and control groups from baseline to endline.

**Results:**

For hand cleanliness, the poorest mothers improved more [Q1 difference in difference, DID: 16% (7, 25%)] than the wealthiest mothers [Q5 DID: 7% (− 4, 17%)]. The poorest households had largest improvements for observed presence of water and soap in handwashing station [Q1 DID: 82% (75, 90%)] compared to the wealthiest households [Q5 DID: 39% (30, 50%)]. Similarly, poorer household demonstrated greater reductions in visible feces on latrine slab or floor [Q1DID, − 25% (− 35, − 15) Q2: − 34% (− 44, − 23%)] than the wealthiest household [Q5 DID: − 1% (− 11, 8%). For reported last child defecation in potty or toilet, the poorest mothers showed greater improvement [Q1–4 DID: 50–54% (44, 60%)] than the wealthier mothers [Q5 DID: 39% (31, 46%).

**Conclusion:**

By simultaneously addressing supply and demand-constraints among the poorest, we observed substantial overall improvements in equity. Within scaled-up programs, a separate targeted strategy that relaxes constraints for the poorest can improve the equity of a program.

**Trial registration:**

WASH Benefits Bangladesh: ClinicalTrials.gov, identifier: NCT01590095. Date of registration: April 30, 2012 ‘Retrospectively registered’.

**Supplementary Information:**

The online version contains supplementary material available at 10.1186/s12939-020-01353-7.

## Background

Despite overall health improvements occurring globally, health remains unevenly distributed with poorer populations having worse health than wealthier populations [1, 2]. Public health interventions can further widen the health gap if they produce greater benefit among wealthier populations, while vulnerable populations in poorer quintiles miss out on the health improvements [3, 4].

In low- and middle-income countries (LMICs), the burden of illnesses fall disproportionately on the poorest and illness associated costs represent a higher proportion of income for the poorest relative to the wealthiest [5]. The poorest are also least likely to receive preventive interventions or have access to life-saving treatments [6]. Targeting the poorest can also be cost-effective. Poorest children are likely to contribute more pathogens to the environment than wealthier children [7]. A recent report found that the investment of $1 through public health interventions averts 1.8 times more deaths among the poor compared with an investment directed at the non-poor [8]. Hence, exploring which intervention components reach the poorest and least educated are key aspects of determining the public health impact of interventions.

Globally, an estimated 1.6million deaths and 105 million disability-adjusted life years (DALY) are attributable to inadequate water, sanitation and hygiene (WASH) [9]. In nearly all LMICs, the poorest have the lowest levels of WASH conditions such as lowest sanitation coverage and the lowest levels of improved water supply coverage [10, 11]. In Bangladesh, the wealthiest households have more than three-fold higher access to adequate sanitation than the poorest households [12]. The poorest and the least educated are also less likely to have soap and water at a dedicated handwashing location and less frequently practice handwashing than their wealthier and more educated counterparts [13–16]. Wealthier and more educated households are more likely to practice microbiologically effective household water treatment [17, 18].

Supply-driven WASH interventions prioritize the provision of WASH infrastructure. However, without adequate behavior change promotion, supply-driven efforts, such as building or subsidizing latrine construction, may be ineffective and unsustainable [19–21]. Residents of many communities in India preferred defecating in the open over using latrines, even when they had access to one [22, 23]. In sub-Saharan Africa, around a quarter of handpumps, which are a major source of drinking water, are not functional due to lack of maintenance [24]. Supply-driven approaches can increase inequity by benefitting the physically and politically connected who are more likely to receive the supplies and more able to arrange maintenance [19].

Another segment of WASH interventions emphasize demand-based approaches. Scaled-up programs in the WASH sector, such as community-led total sanitation (CLTS) and social marketing are examples of strategies that have gained prominence in the recent decades and have emphasized demand-generation effort rather than alleviating supply constraints. CLTS programs place complete responsibility of construction of latrines on the community, and this demand-driven behavior promotion approach has achieved high levels of coverage in studies in Asia and Africa [25–27]. It may be effective on the whole, but it is not clear if this strategy is equitable. For example, as part of CLTS in Indonesia, the poorer households were the least likely to improve their sanitation conditions [28]. Sanitation evaluations elsewhere have also shown how the last people to adopt an intervention are the poorest and least educated [29–33]. Social marketing is another demand-driven strategy applying commercial marketing techniques to promote public health products and behaviors. This strategy can also disproportionately benefit the least poor who can more easily access better products to improve their behaviors [34–38]. In Kenya for example, social marketing of inexpensive chlorine water treatment product resulted in good product penetration and use, but the poorest and least educated were least likely to benefit from this intervention [39].

There is a difference in household level effort required to manage municipality supplied running water that comes directly to the house through taps and toilets connected to municipal sewage systems and thus removes feces from the environment in contrast to a household level system for water and fecal management that has no public component. Household-level WASH interventions, whether supply or demand driven, can put enormous burden on the poorest households by expecting them to purchase all of their infrastructure and supplies and to practice demanding behaviors every day. Supply-driven programs that have not been closely connected to community demand and demand-driven programs that are weak on the supply end both risk worsening inequity. Understanding patterns of uptake of behaviors among the poorest under ideal experimental conditions, such as those of an efficacy trial, can help identify strategies that could be strengthened in routine programmatic conditions for more equitable uptake.

The intervention in the WASH Benefits Bangladesh trial addressed both demand and supply-side constraints. The trial evaluated the health impact of different WASH and nutrition interventions [40]. Since WASH Benefits Bangladesh was an efficacy trial the implementation team aimed to maximize intervention fidelity, access and uptake [41, 42]. The project team provided WASH hardware (enabling hardware such handwashing stations and latrines) free-of-cost and ensured maintenance services. This was coupled with interpersonal behavior change activities by community health promoters (CHPs). The CHPs promoted behavioral recommendations and associated messages developed from the IBM-WASH model [43], to generate demand for WASH behaviors among the target audience [40, 41, 44].CHPs made frequent visit to all households promoting the health behavior messages and troubleshooting WASH hardware-related problems that the participants faced.

After 2 years of WASH Benefits intervention, mean uptake of sanitation and handwashing practices were substantially higher among intervention communities compared to controls [44]. However, improvements in mean levels can mask wealth disparities [45]. We hypothesized that, the least educated and poorest populations had the same level of improvement over time as those better-off. Therefore, the analysis presented in this paper aimed to determine whether uptake of handwashing and sanitation behaviors was equitable across different wealth and education levels.

## Methods

### Study setting and population

The WASH Benefits Bangladesh trial was a cluster randomized trial conducted in rural villages of four districts (Gazipur, Kishoreganj, Mymensingh, Tangail) in central Bangladesh [40, 46]. The study population consisted of pregnant women in their first two trimesters of pregnancy and their children who were expected to be born within approximately 6 months of the survey conducted at baseline. The selection criteria were not based on their wealth. The enrollment began in May 2012 and the study enrolled a total of 5551 pregnant women in 720 geographic clusters.

### WASH benefits intervention

The details of WASH Benefits study methods are described elsewhere [40, 46].WASH Benefits identified 8 mothers living in close proximity to each other, to form a cluster and then formed eight geographical clusters that were grouped to form a block. The study comprised of 90 blocks (720 clusters) in total (participants enrolment and retention summary details in supplementary Table 1). The trial randomly allocated the group of eight geographically proximate clusters to one of the eight study arms that included two control and six intervention arms (Supplementary Table 2): water (W) arm, sanitation (S) arm, handwashing (H) arm, nutrition (N) arm, water, sanitation and handwashing combined arm (WSH) and water, sanitation handwashing and nutrition combined arm (WSHN). We established a buffer zone of a minimum of 15 min walking distance that separated two adjacent clusters so that the intervention would not spillover from cluster to another.

Household from the intervention arms received WASH technologies and nutrition supplements. Supplementary Table 1 details the technological supplies and key behavioral messages for intervention households in the WASH Benefits Trial. For the purposes of the current paper, we analyzed data from only the arms that had sanitation and handwashing interventions (single or combined). The details regarding water treatment and nutrition interventions are described elsewhere [40, 46]. The technologies provided in the sanitation arm included hardware such as concrete ring-based dual pit latrines that had a slab, water seal and walls for privacy. The sanitation intervention also included a potty for young children and a sani-scoop for removal of child feces from the environment to safe disposal in the latrine. The handwashing intervention included the provision of a handwashing station, soapy water bottle and detergent—one station was provided for the latrine and another for the kitchen area. The intervention was implemented in phases over 24 months. In the control arm no intervention and behavioral change messages were delivered.

The intervention also included communication to promote adoption of the behavioral recommendations delivered with high fidelity, by local community health promoters (CHPs) [41]. CHPs were nominated by community members from the same locality to enhance acceptability at household level. Each intervention cluster had its own CHP. Intensive training of CHPs was conducted at the beginning of the project followed by periodical refreshers training throughout the intervention period.

CHPs encouraged regular use of the hardware components through regular household visits, approximately twice a week. The behavioral recommendations were to treat drinking water for children aged < 36 months, use latrines for defecation and the removal of human and animal feces from the compound, wash hands with soap at critical times around food preparation, defecation, and contact with feces.

### Data collection

Hardware presence, functionality and behavioral uptake were measured as part of assessment at baseline (conducted immediately after the enrolment of the pregnant women), midline (end of the first year of intervention) and endline (end of the second year of intervention when the child age was about 21–30 month). Baseline data collection was conducted from May 2012 to July 2013, and endline data collection was conducted from November 2014 to November 2015. Assessment visits were unannounced prior to the individual household visits.

Assessment tools were designedbased on the project indicators which were developed, piloted and revised through substantial feedback, comments and discussions with national and international experts [41]. The field workers collected data based on self-reports and objective observations.

### Data analysis

We analysed three observed and one reported uptake indicators collected during baseline and compared with endline behaviours. First, the trained enumerators collected data on observed hand cleanliness of mothers since contaminated hands. Second, they collected data on presence of water and soap in the handwashing station. Third, they collected data on visible feces and lastly they collected self-reported data on whether the child defecated in a potty or latrine as a measure of potty-use.

For analyses of handwashing variables, we merged the data from all handwashing intervention arms (H, WSH, N + WSH) and for analyses of sanitation variables, we merged all sanitation arms (S, WSH, N + WSH). We generated descriptive summaries for socio-demographic characteristics of our respondents by study arms and wealth quintiles and education levels. We computed the wealth using principal component analysis (PCA) based on household characteristics and ownership of assets. Then we generated five wealth quintiles (Q1-Q5) where Q1 was the lowest and Q5 the wealthiest quintile. We compared observed and reported respondent practices between baseline and endline across wealth quintiles. We also compared observed and reported respondent practices between baseline and endline across education levels. We calculated difference in difference (DID) using generalized linear models (GLM) to compare changes from baseline to endline between households receiving interventions with the control group and adjusted for clustering using a clustered sandwich estimator. In addition, we conducted a three-way interaction between intervention, time and socio-demographic indicators using GLM to directly assess if the impact of the interventions were modified by maternal education and wealth quintiles.

## Protection of study participants

Respondents in the enrolled households (children’s mothers/caregivers) provided informed consent before interviews. The study protocol was reviewed and approved by human subject committees at the International Centre for Diarrhoeal Disease Research, Bangladesh (icddr,b), (PR#11063), University of California, Berkeley (2011-09-3652), and Stanford University (25863).

## Results

The intervention and control arms had similar socio-demographic characteristics (Table 1). However, these characteristics differed between wealth quintiles (Table 2). In Q1, approximately 32% of mothers lacked any formal education while there were only 2.4% in Q5. The mean monthly household income increased across the wealth quintiles (mean (SD) Q1:73(1.1) & Q5: $252(5)). Only 18% of households had electricity in Q1 while more than 90% of the household had this facility in Q5. Among Q1–3, no families had refrigerator whereas 38% of households from Q5 reported refrigerator facilities. Around 27% of the household from the highest quintile (Q5) owned a motorcycle where none had among the first two quintiles (Table 2).

**Table 1:** Baseline characteristics of participants and households according to study arms

Characteristics	n(%) or mean ± SD				
	Control	Sanitation	Handwashing	W + S + H^a^	W + S + H + N^b^
	***N = 1382***	***N = 696***	***N = 688***	***N = 703***	***N = 686***
Maternal age	23.5 ± 5.0	23.7 ± 5.2	23.8 ± 5.4	24.3 ± 5.5	23.8 ± 5.5
Maternal education					
No education	206 (15)	115 (17)	101 (15)	100 (14)	116 (17)
Up-to primary	440 (32)	218 (31)	221 (32)	223 (32)	225 (33)
Above primary	736 (53)	363 (52)	366 (53)	380 (54)	345 (50)
Paternal education					
No education	406 (30)	209 (30)	221 (32)	221 (32)	203 (30)
Up to Primary	412 (30)	204 (29)	211 (31)	198 (28)	228 (33)
Above primary	560 (41)	282 (41)	255 (37)	281 (40)	254 (37)
Monthly household income (US$)	133 ± 2.8	131 ± 3.8	127 ± 3.6	140 ± 4.2	137 ± 4.1
People/household	4.7 ± .06	4.7 ± .08	4.7 ± .08	4.7 ± .08	4.7 ± .08
Children< 3 years /household	0.2 ± .01	0.2 ± .02	0.2 ± .02	0.2 ± .02	0.2 ± .02
Children< 3 years /compound	0.7 ± .02	0.6 ± .03	0.7 ± .03	0.7 ± .03	0.7 ± .03
Own home	1357 (98)	691 (99)	676 (98)	686 (98)	670 (98)
Own latrine	1321 (96)	664 (95)	656 (95)	670 (95)	662 (97)
Had a child potty	61 (4)	28 (4)	35 (5)	27 (4)	30 (4)
Hectares of owned agricultural land (mean ± SD)	.427 ± .025	.407 ± .029	.411 ± .030	.420 ± .028	.459 ± .054
Household have own(%) n	*N* = 1382	*N* = 696	*N* = 688	*N* = 703	*N* = 686
Electricity	784 (57)	408 (59)	405 (59)	426 (61)	412 (60)
Refrigerator	116 (8.4)	57 (8.2)	50 (7.3)	54 (7.7)	52 (7.6)
Mobile phone	1188 (86)	591 (85)	582 (85)	600 (85)	593 (86)
Television	416 (30)	225 (32)	210 (31)	187 (27)	203 (30)
Motorcycle	100 (7.2)	47 (6.8)	35 (5.1)	53 (7.5)	32 (4.7)

^a^W + S + H: in combination of water, sanitation, and hygiene intervention

^**b**^W + S + H + N: in combination of water, sanitation hygiene and nutrition intervention

**Table 2:** Baseline characteristics of participants and households according to wealth quintiles

Characteristics	n (%) or mean ± SD				
	Lowest quintile (Q1)	2nd quintile (Q2)	3^rd^quintile (Q3)	4^th^quintile (Q4)	Highest quintile (Q5)
	***N = 844***	***N = 820***	***N = 831***	***N = 836***	***N = 824***
Maternal age	23.8 ± 5.3	24.5 ± 5.3	24.3 ± 5.4	23.5 ± 5.4	22.8 ± 4.8
Maternal education					
No education	267 (32)	206 (25)	93 (11)	52 (6.2)	20 (2.4)
Up-to primary	374 (44)	318 (39)	314 (38)	229 (27)	92 (11)
Above primary	203 (24)	296 (36)	424 (51)	555 (66)	712 (86)
Paternal education					
No education	486 (58)	354 (43)	252 (30)	129 (15)	39 (4.7)
Up to Primary	264 (31)	285 (35)	293 (35)	281 (34)	130 (16)
Above primary	92 (11)	179 (22)	283 (34)	424 (51)	654 (79)
Monthly household income (US$)	73 ± 1.1	88 ± 1.5	108 ± 1.9	147 ± 3.1	252 ± 5.0
People/Household	3.5 ± 0.04	4.0 ± 0.05	4.4 ± 0.06	5.1 ± 0.07	6.5 ± 0.09
Children< 3 years /household	0.22 ± 0.014	0.20 ± 0.014	0.18 ± 0.014	0.21 ± 0.015	0.29 ± 0.014
Children< 3 years /compound	0.82 ± 0.03	0.74 ± 0.03	0.64 ± 0.03	0.59 ± 0.03	0.55 ± 0.03
Own home	803 (95)	803 (98)	822 (99)	831 (99)	821 (100)
Own latrine	757 (90)	774 (94)	803 (97)	818 (98)	821 (100)
Had a child potty	13 (1.5)	10 (1.2)	18 (2.2)	51 (6.1)	89 (11)
Hectares of owned agricultural land (mean ± SD)	0.13 ± 0.014	0.17 ± 0.011	0.22 ± 0.010	0.38 ± 0.017	0.80 ± 0.039
Household have own n (%)					
Electricity	151 (18)	369 (45)	521 (63)	643 (77)	751 (91)
Refrigerator	0 (0)	0 (0)	0 (0)	18 (2.2)	311 (38)
Mobile phone	476 (56)	673 (82)	778 (94)	808 (97)	819 (99)
Television	5 (0.6)	51 (6.2)	201 (24)	362 (43)	622 (75)
Motorcycle	0 (0)	0 (0)	5 (0.6)	42 (5.0)	220 (26.7)

For observed hand cleanliness, all mothers across education levels, and wealth status showed marked improvement from baseline to endline in the handwashing intervention groups. However, the largest improvements occurred among the youngest mothers compared to older mothers (DID: 12%, CI: 1, 23%), uneducated mothers (DID: 17%, CI: 7, 27%) compared to mothers having some level of primary (DID: 8%, CI: 1, 15%) and above primary education (DID: 10%, CI: 4, 17%, and poorest mothers (Q1-DID: 16%, CI: 7, 25%) compared to mothers from richer quintiles (Table 3).

**Table 3:** Observed handwashing behavior sat baseline and endline, stratified by maternal education and wealth quintiles

Baseline characteristics	Baseline		Endline		Difference in difference (DID)	95% CI	Overall treatment effect	95% CI	Impact of modifier on the treatment effect on outcomes^b^
	Control n (%)	Intervention^a^ n (%)	Control n (%)	Intervention^a^ n (%)					
*Indicator 1: Observed mother’s hand cleanliness (both hands)*							11%	6, 15	
Education of mother									
Uneducated	43 (21)	50 (16)	39 (19)	98 (31)	17%	7, 27			
Some primary education	103 (23)	154 (23)	115 (26)	228 (34)	8%	1, 16			0.0034
Above primary	237 (32)	350 (32)	271 (37)	514 (47)	10%	4, 17			
Wealth index									
Lowest quintile (Q1)	63 (23)	86 (20.6)	52 (19)	137 (33)	16%	7, 25			
2^nd^quintile (Q2)	65 (23)	91 (22)	71 (23)	145 (36)	11%	2, 20			
3^rd^quintile (Q3)	63 (24)	106 (24)	75 (29)	188 (42)	14%	4, 23			0.0041
4^th^quintile (Q4)	84 (30)	111 (27)	102 (37)	161 (39)	5%	−5, 16			
Highest quintile (Q5)	108 (37)	160 (42)	125 (43)	209 (54)	7%	−4, 17			
*Indicator 2: Observed presence of water and soap in household handwashing station (s)*							69%	61, 74	
Education of mother									
Uneducated	27 (13)	41 (13)	20 (12)	242 (96)	84%	75, 90			
Some primary education	75 (17)	97 (15)	77 (21)	537 (98)	79%	75, 85			< 0.0001
Above primary	208 (28)	316 (29)	235 (39)	919 (98)	58%	50, 65			
Wealth index									
Lowest quintile (Q1)	18 (7)	33 (8)	25 (11)	309 (95)	82%	75, 90			
2^nd^quintile (Q2)	35 (12)	49 (12)	37 (16)	336 (98)	83%	75, 90			
3^rd^quintile (Q3)	49 (19)	72 (16)	41 (19)	371 (97)	81%	75, 90			< 0.0001
4^th^quintile (Q4)	69 (25)	109 (26)	90 (38)	351 (98)	58%	50, 70			
Highest quintile (Q5)	139 (48)	191 (50)	139 (58)	331 (99)	39%	30, 50			

^a^All handwashing intervention arms (H, WSH and WSHN)

^b^3-way interaction *p*-value (taking higher order)

Over 95% of all intervention households had water and soap present near handwashing station at endline (Table 3). The greatest improvements from baseline to endline for the presence of soap and water occurred among uneducated mothers compared to educated mothers (DID: 84%, CI: 75, 90%) and the poorest mothers (Q1) compared to richer mothers (Q5-DID: 82%, CI: 75, 90%). We found that both maternal education and wealth quintiles modified the impact of the intervention on mother’s hand cleanliness (*p* < 0.05) and presence of soap and water at the handwashing station (*p* < 0.05).

Visible feces on latrine slab or floors decreased for all wealth groups in the intervention households from baseline to endline. Among all quintiles, observed visible feces on latrine slabs was less common at endline (− 12 to − 13%) than baseline (31 to 58%); a significant reduction occurred in the lowest two quintiles (Q1-DID: -25%, CI: − 35,-15%; Q2-DID: -34%, CI: − 44, − 23%) compared to households from the wealthiest quintile (DID: -1%, CI: − 11, 8%) (Table 4).

**Table 4:** Observed and reported sanitation behavior at baseline and endline, stratified by maternal education and wealth quintiles

Baseline characteristics	Baseline		Endline		Difference in difference (DID)	95% CI	Overall treatment effect	95% CI	Impact of modifier on the treatment effect on outcomes^b^
	Control n (%)	Intervention^a^ n (%)	Control n (%)	Intervention^a^ n (%)					
*Indicator 3: Observed visible feces on latrine slab or floor*							−21%	−26, −16	
Education of mother									
Uneducated	113 (56)	164 (52)	71 (35.2)	48 (14)	−16%	−28, −05			
Some primary education	230 (53)	359 (56)	167 (38.5)	75 (11)	−31%	−39, −22			0.0001
Above primary	339 (46)	496 (46)	235 (32.0)	151 (14)	−18%	−24, −11			
Wealth index									
Lowest quintile (Q1)	150 (56)	239 (58)	96 (35.4)	56 (13)	−25%	−35, −15			
2^nd^quintile (Q2)	141 (51)	205 (54)	118 (42.8)	49 (12)	−34%	−44, −23			
3^rd^quintile (Q3)	153 (59)	227 (55)	94 (36.6)	56 (13)	−19%	−30, −09			< 0.0001
4^th^quintile (Q4)	123 (45)	221 (53)	97 (35.1)	57 (13)	−30%	−40, −20			
Highest quintile (Q5)	115 (40)	127 (31)	68 (23.5)	56 (14)	−01%	−11, 08			
*Indicator 4: Reported last child defecation in potty or toilet among under 3 children*							49%	46, 52	
Education of mother									
Uneducated	1 (0.49)	2 (0.6)	5 (2.4)	187 (56)	54%	48, 60			
Some primary education	4 (1.0)	6 (1.0)	13 (2.9)	365 (55)	52%	48, 56			< 0.0001
Above primary	15 (2.0)	13 (1.2)	92 (12)	627 (58)	46%	42, 50			
Wealth index									
Lowest quintile (Q1)	1 (0.4)	2 (0.5)	7 (2.5)	224 (52)	50%	44, 55			
2^nd^quintile (Q2)	3 (1.0)	1 (0.2)	12 (4.3)	221 (55)	52%	46, 58			
3^rd^quintile (Q3)	0	1 (0.2)	16 (6.1)	246 (58)	52%	46, 58			< 0.0001
4^th^quintile (Q4)	6 (2.2)	9 (2.1)	17 (6.2)	255 (60)	54%	48, 60			
Highest quintile (Q5)	10 (3.4)	8 (2.0)	58 (20)	233 (57)	39%	31, 46			

^a^All sanitation intervention arms (S, WSH and WSHN)

^b^3-way interaction *p*-value (taking higher order)

For all sanitation intervention households, mothers were more likely to report that their child defecated in potty at endline compared to baseline. Mothers having some primary education showed the greatest improvement (DID: 31%, CI: 39, 22%) compared to mothers with no education and mothers with above primary education level. Improvements across all wealth quintiles were similar, with the lowest 4 quintiles showing the greatest improvement compared to the highest quintile (Table 4). Again, in adjusted models, we found that both maternal education and wealth quintiles significantly modified the impact of the intervention on these sanitation indicators (*p* < 0.05).

## Discussion

In rural Bangladesh, when free-of cost WASH technologies were provided along with behavior change promotion, poorer households exhibited the greatest improvement in handwashing and sanitation uptake compared with wealthier households. This finding is consistent to results found in a non-randomized trial in rural Bangladesh, where households with the highest handwashing uptake, as evidenced by presence of low-cost soap alternative, were the ones that received handwashing promotion along with some sort of products and equipment support versus households that received handwashing promotion only [47].

The least educated households in the current study also exhibited greater improvements in handwashing and sanitation uptake compared with more educated households. This suggests that education level was less important for uptake of these preventive interventions, than lack of access to appropriate hardware and encouragement to use the hardware.

In this study, we report that the impacts of interventions were modified by maternal education and measures of socio- economic status. At baseline for both intervention and control groups, the poorest and the groups with the least education had the lowest levels of sanitation and handwashing behaviors and the wealthiest already had relatively higher levels. This distribution is typical for WASH indicators. The poorest and least educated improved the most because they also had the greatest scope for improvement. This pattern is also found in program context— communities with lowest levels of sanitation coverage have the highest improvements, while communities with already high baseline levels of coverage improve the least [27, 48]. Regardless of the degree of improvement over the intervention period for each wealth and education level, the final uptake rates for all wealth and education levels were high. These final uptake levels are higher than handwashing prevalence and latrine use rates recorded in most other handwashing and sanitation interventions reviews [48, 49].

Strengthening the supply-side of an intervention means ensuring access to appropriate hardware such as handwashing stations and durable and functional latrines. In Bangladesh, having a permanent designated handwashing place with soap and water present at that location, was associated with increased handwashing practices independent of socioeconomic status [13]. Furthermore, in many program contexts easy access to hardware does not negatively affect usage. In Zambia, people who paid lower prices for point-of-use water treatment solution were not less likely to use it relative to people who paid higher prices [50]. Similarly, in Kenya, women who received free bednets were equally likely to use them as women who paid for bednets [51].

Uptake is adversely affected when hardware support is suboptimal and scaled-up demand-driven or supply-driven programs are unable to ensure high quality infrastructure. Programs like CLTS may have improved coverage, but improved coverage may not equate to improved levels of usage in the context of large-scale programs [48]. The poorest commonly revert back to open defecation due to low-quality sanitation structures that are prone to collapse, or fill with no provision for emptying [52–54]. Many latrines built as part of CLTS are unimproved and shared; they do not effectively remove waste from the environment and are not sustainable [55].

In settings where the poorest lack financial resources to access health improving hardware, addressing supply constraints through free provision can support a pro-equity impact. However, free distribution may not always be possible and so subsidies [56–59] are one way of alleviating supply constraints and increasing demand among the poorest in scaled-up contexts. Subsidies or free provision of hardware can help the poorest move up the sanitation ladder [55].CLTS programs are increasingly exploring potential of financing mechanisms in order to reach the poorest and most marginalized [53, 54]. An experimental study conducted in Bangladesh compared hygienic latrine ownership between landless poor who were provided community motivation alone, supply-side market intervention alone, and subsidies [60]. The group that was provided subsidies increased latrine ownership the most.

Strengthening the supply side of an intervention should not come at the cost of a weak approach to demand generation. Ensuring access to appropriate WASH hardware is a necessary, but generally not sufficient condition for uptake among the poorest. Behavior change promotion is also important to ensure continued use and habit formation for WASH behaviors.

There are important limitations to the conclusions that we can draw from this analysis. First, the study design does not allow us to specify which component of the WASH Benefits intervention design, the behavior-change promotional activities or the WASH hardware provision, was more important to address equity. Although this is likely to vary by context, future studies could deploy experimental methods with varying levels of demand and supply-side components to understand which strategy would be most cost-effective in order to achieve equitable impact.

A second limitation is that the current analysis examined wealth and education inequity only. However, considerations of inequity outside the controlled setting of a randomized controlled trial may also affect communities including geographical, ethnic and gender inequity [61].

Finally, the WASH Benefits Bangladesh intervention occurred as part of an intensive scientific study with focused effort to maximize uptake. It is unclear whether interventions with the intensity typical of WASH programs could generate similar pro equity results. High level of CHP engagement in smaller clusters, and free or heavily subsidized provision of hardware to all participants are not scalable in most circumstances. Within scaled-up programs, one possible approach could be to have two distinct strategies—one focused on efficiency to reach the highest number of program beneficiaries, and a separate dedicated strategy for the poorest that can mimic the conditions of an efficacy trial. Such as approach has been employed by BRAC WASH program, where all participants received sanitation promotion, but the ultra-poor are provided free latrines once 80% coverage is reached in a community, and in areas where coverage is less than 80%, the poor and ultra-poor are provided with subsidies [62]. Such a strategy is likely to be more effective in terms of improving equity of a program.

## Conclusion

WASH interventions, whether supply-driven or demand-driven, have often adopted a strategy, whereby the people easiest to reach, the wealthier and the more educated populations, benefitted from coverage. However, this strategy has been inequitable. The WASH Benefits trial found that given sufficient support, the most vulnerable populations exhibit the greatest benefit from WASH interventions. What works for efficiency, that is reaching the greatest number of people with an intervention, may not work for equity, which requires reaching the most vulnerable. There are obvious trade-offs between health benefits, cost-effectiveness and cost-recovery of WASH technologies [63]. While it is not possible to deploy an optimal strategy for all participants in a scale-up WASH program, this strategy of strengthening of the supply and demand sides of the intervention for the most vulnerable populations within a scaled-up program offers the potential for substantial improvements in overall program equity.

## Supplementary Information

**Additional file 1: Supplementary Table 1**. Participants enrolment and retention

**Additional file 2: Supplementary Table 2**. Technological supplies and key behavioral messages for intervention households in the WASH Benefits trial, Bangladesh

## Data Availability

Data can be made available through the authors.

## Abbreviations

LMIC: Low and middle-income countries
DALY: Disability-adjusted life years
WASH: Water, sanitation and hygiene
CLTS: Community led total sanitation
IBM-WASH: Integrated behavioural model-water, sanitation and hygiene
W: Water
S: Sanitation
H: Handwashing
N: Nutrition
WSH: Water, sanitation and handwashing
WASN: Water, sanitation, handwashing and nutrition
CHP: Community health promoter
PCA: Principal component analysis
Q: Quintiles
DID: Difference in difference
GLM: Generalized linear models
