# Supplementary material for: Achieving equitable uptake of handwashing and sanitation by addressing both supply and demand-based constraints: findings from a randomized controlled trial in rural Bangladesh
Source: Int J Equity Health. 2021 Jan 6;20:16. doi: 10.1186/s12939-020-01353-7 (PMC7789645; doi:10.1186/s12939-020-01353-7)
Supplement: Supplementary file 1 — Additional file 1: Supplementary Table 1. Participants enrolment and retention [file 12939_2020_1353_MOESM1_ESM.docx]

Supplementary table 1: Participants enrolment and retention
