# Supplementary material for: Achieving equitable uptake of handwashing and sanitation by addressing both supply and demand-based constraints: findings from a randomized controlled trial in rural Bangladesh
Source: Int J Equity Health. 2021 Jan 6;20:16. doi: 10.1186/s12939-020-01353-7 (PMC7789645; doi:10.1186/s12939-020-01353-7)
Supplement: Supplementary file 2 — Additional file 2: Supplementary Table 2. Technological supplies and key behavioral messages for intervention households in the WASH Benefits trial, Bangladesh [file 12939_2020_1353_MOESM2_ESM.docx]

**Supplementary table 2: technological supplies and key behavioral messages for intervention households in the WASH Benefits trial, Bangladesh**

| **Intervention** | **Hardware packages** | **Key behavioral recommendations** |
| --- | --- | --- |
| Water (W) | Safe water storage vessel  Aquatabs for water treatment | Targeted children drink treated, safely stored water |
| Sanitation (S) | Dual pit latrine  Child potty  Sani-scoop for removal of feces | Family use double pit latrines  Potty use for child defecation.  Safely dispose of feces into latrine or pit |
| Handwashing (H) | Designated handwashing station for latrine &kitchen  Soapy water bottle with detergent | Wash hands with soap:  -after defecation  -after cleaning a child’s anus  -during food preparation |
| Nutrition (N) | Exclusive breastfeeding up to 6 months  Lipid based nutrient supplement (LNS) for children aged 6-24 months | Exclusive breastfeeding up to 180 days  Introduce diverse complementary food at 6 months.  LNS from 6–24 months |
| Combined (WSH) | Combination of water, sanitation & handwashing intervention | Same message as those for the water, sanitation, and handwashing interventions |
| Combined (N+WSH) | Combination of WSH with nutrition intervention | Same as water, sanitation handwashing and nutrition interventions |
